# Quality of Life After Early Clot Removal for Acute Iliofemoral Deep Vein Thrombosis^☆^

**DOI:** 10.1016/j.ejvsvf.2023.12.004

**Published:** 2024-01-04

**Authors:** Jay M. Bakas, Catherine van Montfrans, Adriaan Moelker, Renate R. van den Bos, Wendy S.J. Malskat, Hence J.M. Verhagen, Marie Josee E. van Rijn

**Affiliations:** a Department of Vascular and Endovascular Surgery, Erasmus Medical Centre, Rotterdam, the Netherlands; b Department of Dermatology, Erasmus Medical Centre, Rotterdam, the Netherlands; c Department of Radiology and Nuclear Medicine, Erasmus Medical Centre, Rotterdam, the Netherlands

**Keywords:** Deep vein thrombosis: endovenous treatment, Mental health, Patient reported outcomes, Quality of life

## Abstract

**Objective:**

To evaluate patient reported outcome measures after early clot removal for acute deep vein thrombosis (DVT), using the Chronic Venous Disease Quality of Life Questionnaire (CIVIQ-20) and the Short Form Health Survey (SF-36).

**Methods:**

Cross sectional design. Patients who underwent early clot removal between June 2012 and November 2021 were asked to complete the two questionnaires once. Lower CIVIQ-20 and higher SF-36 scores indicate better quality of life (QoL). Primary endpoints were the median scores. The one sample Wilcoxon signed rank test was used to compare SF-36 physical and mental component summary (PCS and MCS) to the normative and CIVIQ-20 to the minimum. Secondary, non-parametric independent t test or Fisher's exact test examined how age, sex, body mass index, stent placement, re-intervention, and time of questionnaire completion related to QoL. Multivariable linear regression tested whether various variables were associated with QoL.

**Results:**

The response rate was 73.5% (*n* = 39). Median time of questionnaire completion was 1.8 years (interquartile range [IQR] 3.1) after clot removal. The median CIVIQ-20 of 29.0 (IQR 28.0) was slightly higher than the minimum value 20.0 (*p* < .001). The median PCS (50.5, IQR 16.6) and median MCS (50.2, IQR 14.2) did not differ from the normative of 50.0. However, wide IQRs indicated impairments for a subgroup of patients. None of the tested variables affected QoL except for the finding that re-interventions had a significantly negative impact on the SF-36 MCS (standardised β coefficient of −0.4, *p* = .030).

**Conclusion:**

Overall patient reported outcome measures were satisfactory after early clot removal, but impaired physical and mental functioning levels were present in a subgroup of patients. Re-interventions were found to have a negative impact on mental QoL. This finding was independent of time that had passed between the procedure and questionnaire completion. This study emphasises that mental functioning deserves attention, besides the widely recognised physical consequences after invasive acute iliofemoral DVT treatment.

## Highlights

• Patient reported outcomes were good after intervention for deep vein thrombosis.
• Impaired physical and mental functioning was present in a subgroup.
• Re-intervention was significantly predictive for impaired mental functioning.
• Quality of life should be assessed after intervention.

## Introduction

Acute deep vein thrombosis (DVT) is diagnosed in one or two per 1 000 persons annually.^1^ Oedema, erythema, and pain in the leg are common features of acute DVT and are more severe if the thrombus extends across the inguinal ligament into the iliac veins. Patients with acute iliofemoral DVT are at higher risk of developing post-thrombotic syndrome (PTS) than patients with acute DVT below the level of the common femoral vein.^2^

Acute DVT has an impact on both physical and mental functioning levels.^3^ The former often receives attention during treatment and follow up, but the latter is more easily overlooked. Physical and mental functioning levels may be severely impaired within the first month after (non-invasively treated) venous thromboembolism (VTE).^4^

The current guidelines of the European Society for Vascular Surgery (ESVS) recommend endovenous treatment in selected patients with symptomatic acute iliofemoral DVT.^5^ This involves early clot removal using pharmacological thrombolysis, standalone percutaneous mechanical thrombectomy (PMT), and or pharmacological catheter directed thrombolysis (PCDT), often followed by stenting. The goal is prevention of PTS and limb salvage in case of phlegmasia cerulea dolens. Although early clot removal has been shown to reduce the risk of PTS,^5^ it may not always improve quality of life (QoL) compared with conservative treatment.^6^ Early clot removal might even serve as an (temporary) extra stressor, because of its invasive character.

This cross sectional study evaluated physical and mental QoL after early clot removal for acute iliofemoral DVT, using the Chronic Venous Disease Quality of Life Questionnaire (CIVIQ-20) and the Short Form Health Survey (SF-36), and searched for risk factors affecting QoL outcome.

## Materials and methods

### Cohort

Patients who underwent early clot removal for acute iliofemoral and or iliocaval DVT were screened retrospectively from the hospital's first procedure in June 2012 until November 2021. Patients with inadequate understanding of the Dutch language, cognitive impairment, or who were impossible to invite due to death or living abroad were excluded. Eligible patients were asked for informed consent to complete two questionnaires: the Chronic Venous Disease Quality of Life Questionnaire (CIVIQ-20) and the Short Form Health Survey (SF-36). Patients with informed consent and completed questionnaires were included. The questionnaires were completed in a cross sectional design (≥3 months after the primary procedure), meaning that the time after the procedure varied for all patients. Non-responders and patients with incomplete questionnaires were contacted once again and excluded if no response was sent back after a minimum interval of three months. This study was approved by the local medical ethics board (MEC-2020-0429).

The following baseline characteristics were collected retrospectively from medical records: age (in years at time of completing the questionnaire), sex, body mass index (BMI) measured during admission for the intervention, previous diagnosis of diabetes mellitus (DM), peripheral arterial occlusive disease (PAOD), and smoking (either current or former). The current study prospectively recorded venous (stent) patency as a component of a larger study into which it was integrated.^7^ Therefore, patients underwent recent assessment of venous (stent) patency with duplex ultrasound (DUS) or computed tomography (CT). Recent venous (stent) patency assessment was missing for one patient, but this patient has remained free of complaints and or re-intervention to date. The Strengthening the Reporting of Observational Studies in Epidemiology (STROBE) guidelines were used to ensure high quality presentation of the study.^8^

### Early clot removal

All included patients underwent early clot removal using pharmacological thrombolysis, standalone PMT, and or PCDT, followed by venous stenting if a >50% stenosis was detected by venography and or intravascular ultrasound. Re-intervention was performed in stent thrombosis cases, stenosis, and or device failure (such as stent fracture). Re-interventions included pharmacological thrombolysis, PMT, PCDT, percutaneous transluminal angioplasty, and or additional stent implantation. Permanent occlusion was defined as a vein and or stent occlusion without further options to re-open it.

### Quality of life

The CIVIQ-20 is a validated 20 question disease specific questionnaire for chronic venous disease (CVD) that assesses lower limb complaints, impact on daily activities, and mental state.^9^^,^^10^ It is divided in four domains: leg pain, physical activity, psychological activity, and social activity. The total score ranges from 20 to 100. A higher score indicates more complaints and a worse QoL.

The SF-36 is a 36 question validated survey that assesses generic QoL across eight domains: physical functioning, role limitations due to physical health (role physical), bodily pain, general health, vitality, social functioning, role limitation due to emotional problems (role emotional), and mental health.^11^ Each domain scores on a scale of 0–100, with a higher score indicating a better QoL, in contrast to the CIVIQ-20 scores. The SF-36 contains a physical and mental component summary (PCS and MCS), which are normative based scores using a mean score of 50 from the general population of the United States as a reference.

### Endpoints

Primary endpoints were total median CIVIQ-20 score and SF-36 PCS and MCS after early clot removal with or without venous stenting for acute iliofemoral DVT. Secondary endpoints were risk factors for QoL outcomes.

### Statistical analysis

Data were analysed using IBM SPSS software version 28.0.1.0. The questionnaire responses and demographics were analysed using descriptive frequencies. Continuous variables are presented as mean with standard deviation (SD), or median with interquartile range (IQR). Additionally, boxplots were used to visualise the patient reported outcome measures (PROMs). The one sample Wilcoxon signed rank test was used to calculate *p* values comparing SF-36 component summaries to the normative value of 50.0 of the general population of the United States. For the CIVIQ-20, the one sample Wilcoxon signed rank test was used to compare the outcomes to the minimum value of 20, indicative of no CVD complaints. The Shapiro–Wilk test was used to test whether samples were normally distributed. Multivariable linear regression was used to evaluate whether age, sex, BMI, stent placement, re-intervention, and time between questionnaire completion and the procedure were significantly associated with QoL. A non-parametric independent t test (continuous variables) or Fisher's exact test (categorical variables) was used to assess these parameters between patients within the first quartile (Q1) and last quartile (Q4) of the CIVIQ-20 and SF-36 PCS and MCS. Statistical significance was set at *p* < .05. Missing data were excluded from the analysis.

## Results

### Cohort

**Figure 1:**
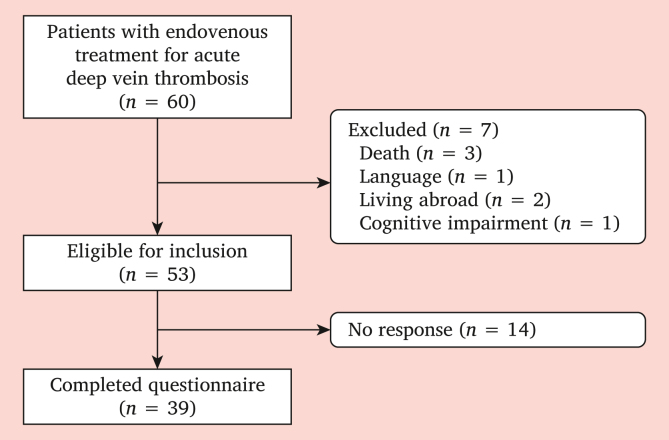
Patient inclusion flowchart.

A total of 60 patients underwent early clot removal for acute iliofemoral DVT, of whom seven were excluded, as shown in Figure. 1. The remaining 53 eligible patients were invited to complete the questionnaires, with a response rate of 73.5% (*n* = 39). Characteristics of the included patients are shown in Table 1. Patients had a mean age of 44 ± 17 years at time of completing the questionnaires and 59% were female. Early clot removal was followed by venous stenting in the majority of patients (87.2%). The median time between intervention and questionnaire completion was 1.8 years (IQR 3.1). Only one patient had permanent occlusion and venous (stent) patency was preserved in all others, 18.4% underwent reintervtion. Data on BMI and smoking were missing for three and five patients, respectively.

**Table 1:** Patient characteristics.

Characteristic	Acute DVT (*n* = 39)
Age – y	44 ± 17
Female	23 (59.0)
Body mass index∗	25.8 (6.6)
Diabetes	1 (2.6)
Peripheral arterial occlusive disease	1 (2.6)
Smoking∗	8 (20.5)
Stented	34 (87.2)
Permanent occlusion	1 (2.6)
Time of questionnaire completion – y	1.8 (3.1)

Data are presented as *n* (%), mean ± standard deviation, or median with interquartile range. Age and follow up calculated at time the questionnaire was completed. Time of questionnaire completion is shown in years between the primary procedure and completion of the questionnaire.

∗ Missing values are *n* = 3 for body mass index and *n* = 5 for smoking.

### Chronic Venous Disease Quality of Life Questionnaire

**Figure 2:**
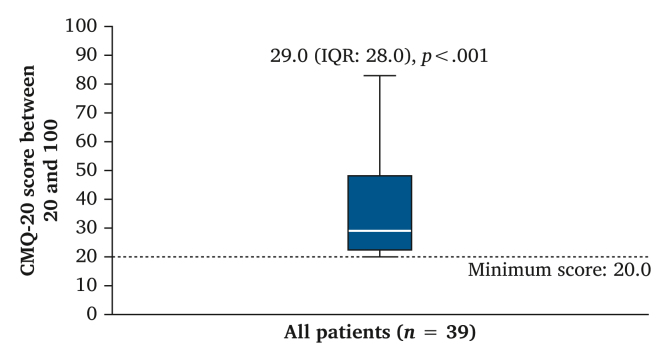
Total Chronic Venous Disease Quality of Life Questionnaire (CIVIQ-20) in all included patients. One sample Wilcoxon signed rank test to compare CIVIQ-20 with the minimum score of 20 indicated by the reference line.

A boxplot with the CIVIQ-20 score is shown in Figure. 2. The minimum score of 20 is the best outcome, indicating no complaints and or disabilities due to CVD. The total cohort had a median score of 29, slightly higher (worse QoL) than the minimum value of 20 (*p* < .001). The interquartile range shows that some patients scored around the minimum score of 20, while the upper border shows the highest score being almost 80.

### Short Form Health Survey component summary scores

**Figure 3:**
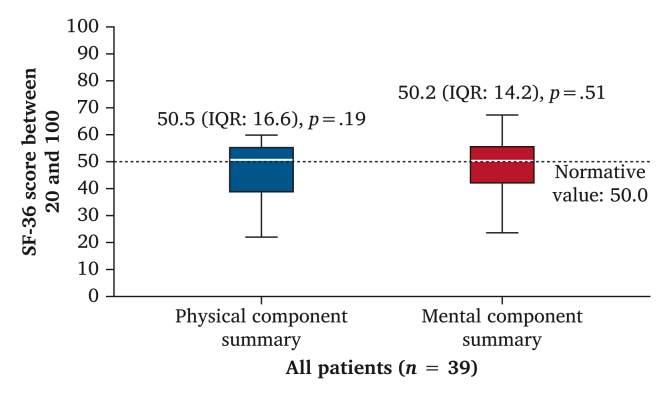
Mental and physical component summaries (Short Form Health Survey [SF-36]) in all included patients. One sample Wilcoxon signed rank test comparing SF-36 scores to the normative value of 50.0 for general population (United States) indicated by the reference line.

A boxplot for the SF-36 component summaries is shown in Figure. 3. The median PCS (50.5, IQR 16.6) and median MCS (50.2, IQR 14.2) were not significantly different from the normative value of 50.0. Meanwhile, the lower and higher borders of the IQRs indicated impaired and better physical and mental functioning levels, respectively, for substantial subgroups of patients.

### Subdomains

An extensive overview the SF-36 and CIVIQ-20 complementary subdomains is provided in Supplementary Table S1. Vitality (median 60.0, IQR 35.0) and General Health Perceptions (median 62.0, IQR 35.0) were the lowest (worst) SF-36 domains. The vitality scale includes four questions focused on energy and complaints of fatigue within the past four weeks. General health perceptions include five questions, focusing on the overall rating of current health in general.

### Long term quality of life

Multivariable regression analysis to test the effect of several factors on QoL outcomes are shown in Supplementary Table S2. Included variables were age, sex, BMI, stent placement, re-intervention, and time between questionnaire completion and the procedure. None of the variables was associated with QoL except that re-interventions had a significant negative impact on the SF-36 MCS. For the CIVIQ-20, this was borderline significant (standardised β coefficients of re-intervention: −0.2 for SF-36 PCS, *p* = .30; −0.4 for SF-36 MCS, *p* = .030; and 0.4 for CIVIQ-20, *p* = .051).

### Subgroup analysis: PROM within the first and last quartile

Table 2 shows the SF-36 for patients within the first (Q1, worst) and last (Q4, best) quartile and for CIVIQ-20 between the last (Q4, worst) and first (Q1, best) outcomes. Age, sex, BMI, stent placement, re-interventions, and time between questionnaire completion and intervention did not affect QoL outcome.

**Table 2:** Characteristics of patients within the worst and best quartile of the Short Form Health Survey (SF-36) mental component summary (MCS), SF-36 physical component summary (PCS), and Chronic Venous Disease Quality of Life Questionnaire (CIVIQ-20).

Characteristic	SF-36 PCS			SF-36 MCS			CIVIQ-20		
	Q1 (*n* = 10)	Q4 (*n* = 10)	*p* value	Q1 (*n* = 10)	Q4 (*n* = 10)	*p* value	Q4 (*n* = 10)	Q1 (*n* = 10)	*p* value
Age – y	40 ± 18	33 ± 16	.28	41 ± 14	45 ± 23	.91	39 ± 18	38 ± 22	.74
Female	6 (60.0)	7 (70.0)	>.99	6 (60.0)	6 (60.0)	>.99	6 (60.0)	5 (50.0)	>.99
Body mass index	26.8 ± 6.8∗	26.7 ± 4.1†	>.99	27.8 ± 5.7	27.3 ± 5.3∗	.66	25.3 ± 6.0∗	26.6 ± 2.6∗	.55
Stented	8 (80.0)	9 (90.0)	>.99	9 (90.0)	10 (100)	>.99	8 (80.0)	9 (90.0)	>.99
Re-intervention	3 (30.0)	1 (10.0)	.58	4 (40.0)	2 (20.0)	.63	3 (30.0)	1 (10.0)	.58
Time of questionnaire completion – y	2.8 ± 2.1	3.4 ± 3.1	.74	2.2 ± 1.7	0.97 ± 1.0	.090	2.1 ± 1.9	2.6 ± 2.9	>.99

Data are shown as mean ± standard deviation, or *n* (%). CIVIQ-20 = Chronic Venous Disease Quality of Life Questionnaire; SF-36 = Short Form Health Survey; Q = quartile; PCS = physical component summary; MCS = mental component summary.

Time of questionnaire completion is shown in years between the primary procedure and completion of the questionnaire.

∗ *n* = 1 missing value excluded.

† *n* = 2 missing values excluded.

### Permanent occlusion

Permanent occlusion was found in one male patient aged 22 years at the time of questionnaire completion (3.1 years after the primary procedure). He was stented for acute DVT from the inferior vena cava into the left common femoral vein. His CIVIQ-20 score was 78, SF-36 PCS 29.3, and SF-36 MCS 25.1. Comorbidities included medical history of multiple venous thrombotic events, thrombophilia, and depression.

## Discussion

Physical and mental QoL measured by CIVIQ-20 and SF-36 was satisfactory at various timepoints after early clot removal for acute iliofemoral DVT in this cohort of 39 included patients. However, a substantial subgroup experienced physical and or mental disabilities following the procedure. Common complaints were related to fatigue and impaired rating of the patients’ own health. This was not explained by a more recent procedure (in terms of longer or shorter time between treatment and questionnaire completion), or by age, sex, BMI, or stenting. However, re-intervention was a significant predictor for lower mental QoL indicated by the SF-36 questionnaire.

Three trials randomised between early clot removal and anticoagulant therapy alone for acute iliofemoral or proximal DVT: ATTRACT trial (Acute Venous Thrombosis: Thrombus Removal with Adjunctive Catheter-Directed Thrombolysis), CAVA-trial (Catheter Versus Anticoagulation Alone for Acute Primary Iliofemoral DVT), and CaVenT-trial (Catheter Directed Venous Thrombolysis in Acute Iliofemoral Vein Thrombosis).^12^, ^13^, ^14^ Differences in SF-36 were reported from baseline until 24 months for the ATTRACT-cohort.^12^ The PCS at one month was higher after early clot removal than anticoagulant therapy alone (difference 3.2, *p* = .001), indicating faster relief of symptoms and signs of acute DVT. No significant differences were found from baseline to other time points, including 24 months of follow up. The MCS improved for both groups over time, but no differences were found between the two treatment strategies from baseline to any assessment, including 24 month follow up. The SF-36 physical health, mental health, and general health domains were reported at 12 months and the final follow up visit for the CAVA cohort.^13^ The intervention group had a (slight) decrease in all domains, indicating a better QoL, but without statistical significance. Statistically significant differences were found between the treatment arms for physical health: additional thrombolysis scored 2.7 points lower, while standard treatment scored 4.4 points higher (*p* = .048), indicating early clot removal to be beneficial for physical functioning. Other PROMs (EQ-5D and VEINES-QOL/Sym) were reported from the CaVenT cohort, without differences in long term QoL between patients with acute DVT who underwent additional thrombolysis and who received standard treatment.^14^ However, it is important to note the heterogeneity between studies in selection criteria (e.g., inclusion of femoropopliteal DVT), treatment techniques, and questionnaires used.

In the present study, QoL was overall satisfactory after early clot removal in iliofemoral DVT patients, but impaired in a subgroup. Assessing QoL is the only way to identify these patients in future clinical practice and should be part of routine follow up, even if patients have a patent iliofemoral vein with or without a stent. Awareness of reduced mental health is just as important as physical disability. Depression is a common cause of post-operative morbidity (e.g., pain, infections, and impaired QoL) after various conditions.^15^ Psychological counselling may be useful to improve expectations, mental health, and QoL for patients with acute DVT, and should be studied in the future. Impaired mental functioning levels after acute DVT are in line with current experiences. Recently, peri-procedural mental support was introduced for patients with acute DVT who are selected for early clot removal in this institution through counselling by a psychologist.

While early clot removal may directly resolve acute complaints, it is also a potential extra stressor due to its invasive character, risk of complications, and possible re-interventions. Evidence of a relationship between timing of questionnaire completion and QoL was missing, suggesting that either the procedure does not affect mental health, or that impaired mental health persists, even after several years. Performance of re-intervention was a significant predictor for lower (worse) SF-36 MCS, emphasising the need to be aware of impaired mental health status in these patients and to prevent re-interventions by patient selection, high percentage of clearance of the vein, proper stent placement, strict follow up, and adequate anticoagulation.

This study has several limitations, including that PROMs were measured at different time points after early clot removal, while, on the other hand, this allowed study of the effect of time since procedure and long term effects. The relatively small sample size of the cohort may have influenced the findings by causing either type I errors (false positives), or type II errors (false negatives). For example, the lack of a significant association between re-intervention and CIVIQ-20 might be a false negative finding. This emphasises the need for future studies with bigger sample sizes. While a response rate of 73.5% was reached in this study, it is also unknown whether QoL was worse or better compared with the 26.5% non-responders. The pre-treatment QoL was unknown due to the unexpected nature of the acute DVT, making it impossible to correct for pre-existing mental and or physical impairments.

### Conclusion

The PROMs after early clot removal in acute iliofemoral DVT are overall satisfactory; however, impaired physical and mental functioning levels are present in a subgroup. Re-intervention is a predictor for impaired mental functioning. Absence of a relationship between time of questionnaire completion and QoL suggests that impaired functioning levels may persist over time. These findings emphasise the attention mental functioning deserves, besides the widely recognised attention to physical impairment, after invasive treatment for acute iliofemoral DVT, especially when re-interventions take place. Addressing mental health, fatigue, and general health perception directly after the intervention and during follow up may help to improve patients’ expectations and quality of life.

## Conflict of interests

A.M. and M.R. reported conflicting interests. A.M. is a proctor and speaker for Cook Medical and advisor for Angiocare. M.R. is a speaker for Medtronic and Inari.

## Funding

None.
