# Supplementary material for: Quality of Life After Early Clot Removal for Acute Iliofemoral Deep Vein Thrombosis
Source: EJVES Vasc Forum. 2024 Jan 4;61:44–9. doi: 10.1016/j.ejvsvf.2023.12.004 (PMC10839761; doi:10.1016/j.ejvsvf.2023.12.004)
Supplement: Multimedia component 1 [file mmc1.pdf]

**Supplementary Table S1** Patient-reported outcomes in all acute deep vein thrombosis patients after endovenous treatment

| SF-36 | All patients (n= 39) |
|---|---|
| Physical Functioning (0-100) | 90.0 (IQR: 35.0) |
| Role-Physical (0-100) | 75.0 (IQR: 75.0) |
| Bodily Pain (0-100) | 74.0 (IQR: 49.0) |
| General Health Perception (0-100) | 62.0 (IQR: 35.0) |
| Vitality (0-100) | 60.0 (IQR: 35.0) |
| Social Functioning (0-100) | 87.5 (IQR: 37.5) |
| Role-Emotional (0-100) | 100 (IQR: 66.7) |
| Mental Health (0-100) | 72.0 (IQR: 16.0) |
| Physical Component Summary (0-100; 50.0 = US reference norm) | 50.5 (IQR: 16.6) |
| Mental Component Summary (0-100; 50.0 = US reference norm) | 50.2 (IQR: 14.2) |
| CIVIQ-20 | |
| Leg Pain (question 1-4) | 6.0 (IQR: 7.0) |
| Physical Activity (question 5-7, 9) | 5.0 (IQR: 5.0) |
| Psychological Activity (question 12-20) | 15.0 (IQR: 13.0) |
| Social Activity (question 8, 10, 11) | 4.0 (IQR: 4.0) |
| Total score (20-100) | 29.0 (IQR: 28.0) |
| Time of questionnaire completion *Years* | 1.8 (IQR: 3.1) |

*Time of questionnaire completion is shown in years between the primary procedure and completion of the questionnaire. PCS= physical component summary. MCS= mental component summary. Variables presented as median with interquartile range (IQR). US= United States*

**Supplementary Table S2.** Multivariate regression model for predicting PROMs

| SF-36 PCS (n= 39 patients) | **Unstandardized ß coefficient** ± standard error | **Standardized ß coefficient** | **p-value** |
|---|---|---|---|
| **Constant** | 44.2 ± 13.5 | | .003 |
| **Age** Years | -0.3 ± 0.1 | 0.04 | .85 |
| **Sex** | 3.2 ± 4.3 | 0.1 | .46 |
| **Body Mass Index** | -0.08 ± 0.4 | -0.04 | .85 |
| **Stented** | 4.6 ± 6.5 | 0.1 | .49 |
| **Re-intervention** | -5.4 ± 5.1 | -0.2 | .30 |
| **Time of questionnaire completion** *Years* | -0.02 | -0.1 | .52 |
| SF-36 MCS (n= 39 patients) | **Unstandardized ß coefficient** ± standard error | **Standardized ß coefficient** | **p-value** |
| **Constant** | 48.1 ± 11.4 | | <.001 |
| **Age** Years | -0.01 ± 0.1 | -0.03 | .90 |
| **Sex** | -1.1 ± 3.6 | -0.06 | .76 |
| **Body Mass Index** | 0.1 ± 0.3 | 0.07 | .71 |
| **Stented** | 1.9 ± 5.5 | 0.07 | .74 |
| **Re-intervention** | -9.5 ± 4.3 | -0.4 | .03 |
| **Time of questionnaire completion** *Years* | -0.02 ± 0.02 | -0.2 | .33 |
| CIVIQ-20 (n= 39 patients) | **Unstandardized ß coefficient** | **Standardized ß coefficient** | **p-value** |
| **Constant** | 40.5 ± 21.2 | | .07 |
| **Age** Years | -0.006 ± 0.2 | -0.006 | .98 |
| **Sex** | -0.4 ± 6.7 | -0.01 | .96 |
| **Body Mass Index** | -0.1 ± 0.6 | -0.03 | .84 |
| **Stented** | -9.0 ± 10.3 | -0.2 | .39 |
| **Re-intervention** | 16.2 ± 8.0 | 0.4 | .051 |
| **Time of questionnaire completion** *Years* | 0.05 ± 0.04 | 0.2 | .23 |

*Time of questionnaire completion is shown in years between the primary procedure and completion of the questionnaire. PCS= physical component summary. MCS= mental component summary.*
